# Some Remarks on the Root-Canal Question

**Published:** 1895-01

**Authors:** J. W. Downey

**Affiliations:** Fort Collins, Col.


ARTICLE III.

### SOME REMARKS ON THE ROOT-CANAL QUESTION.

BY J. W. DOWNEY, M. D., D. D. S., FORT COLLINS, COL.

It is not my purpose to present any special pet method of treating and filling the canals of the teeth, but to briefly review some of the methods in vogue and recently advocated by high authority, together with a liberal admixture of the author's personal experience while searching for light.

As the title of the paper would indicate, the subject under consideration is unsettled. The practice of treating and filling the canals of teeth is comparatively new; the very beginning dates about the middle of this century and the practice has become general only within the last few years. Like all unsettled questions in therapeutics, the methods employed have only been limited by the number of practitioners. My first instruction on the subject was as follows: Success in the treatment of root-canals is in proportion to the amount of pulp tissue removed and the completeness of the filling, and this for years has been the accepted orthodox formula. This statement of the case implies that removal of the entire pulp and completely filling the canal would insure perfect success, and, on the other hand, failure to remove the entire pulp and fill the canal completely would be followed sooner or later by failure. Upon this foundation I determined to build the superstructure of my practice in this line, and with the limited skill of a novice and the zeal of a recent convert I attempted the treatment of all canal cases which came to my hands.

The necessities of the community in which I was located gave me a considerable practice, and as I considered my mission on earth to be to save teeth under all conditions and circumstances, my physical endurance was often sorely taxed and I did not always enjoy the satisfaction of an approving conscience, because it soon became apparent that for me, at least, it was impossible to reach the apex of some roots through the canal. This I charged to lack of skill, thinking that all other dentists measured up to the ideal standard heretofore mentioned, till finally, while conversing on the subject with a very skillful operator in whom I reposed the utmost confidence, he let fall this remark: "You will find that nature is very kind in these cases; she will in great measure make amends for our imperfect work." "What!" said I, "you do not dare to leave any of the pulp in a canal before filling?" "Oh, yes; it is quite impossible to remove all of the pulp in many cases." Shortly afterward, while discussing the buccal canals of upper molars with another gentleman of equal skill and longer service in the profession, I had another stone removed from my ideal foundation. Said he: "If you remove all of the pulp except that part contained in buccal canals, sterilize and fill the palatal canal, you need not worry about the case." The remarks of these gentlemen had a very soothing effect on my conscience, for, as a matter of fact, my practice all along had been in accordance with these precepts.

Some years afterwards the subject of retaining pulp tissue came up in one of the old Eastern societies. I have not been able to find the record, but I think it was one of the New York district societies. Many of the giants of the East were present and they almost unanimously stood up like brave men and acknowledged that they were frequently obliged to allow pulp tissue to remain in the canals; and further, that no serious results were anticipated. And now come Herbst, of Bremen, and Bodecker, of New York, and declare that all of the pulp contained in the roots may be retained. The fact that there is such a multiplicity of methods advocated for the treatment of this class of cases is sufficient evidence that the question is not settled. The materia medica has been ransacked for specifices and a multitude of materials have done service as root-fillings, yet no man can point to a method and declare, "Herein lies perfect success."

The introduction into dental practice of the methods of antiseptic surgery has greatly simplified the matter, but the dental profession has not yet unanimously adopted these methods. Some good practitioners still treat abscesses by drilling into the canal and leaving it open for a few days; others dispense with the rubber dam during the entire treatment. The immediate fillers have had their day, and now those who pin their faith to diffusible antiseptic drugs, with time as an element in the case, seem to be in the lead. Harlan, of Chicago, who appears to hold the championship in this school, no doubt regards the Herbst method as supremely ridiculous; while, on the other hand, Herbst and his followers consider their antagonists as overzealous cranks who are daily subjecting themselves and their patients to unnecessary expenditure of physical force. Miller, of Berlin, takes middle ground by advocating a method of pickling pulps, not easily removed, but he is not enthusiastic in his recommendation of the method, since the difficult task of reaching remote portions of the canal has not been overcome.

And now what are the conclusions to be drawn from the imperfect review of a very important subject?

1st. That we need a Moses to lead us out of bondage of doubt and uncertainty.

2d. Possibly Herbst is such a leader.

3d. There should be some systemic, concerted action by dental societies to test the method; because few men will have the courage to adopt a treatment so radically different from methods established by years of usage and the practically unanimous approval of the entire profession, unless they have the moral support of their fellow-practitioners. When Herbst's method shall have been weighed in the balance of time and not found wanting, our labors in the operative department will be reduced about one fourth. The saving to our patients in time, expense and comfort will be immense. In view of these manifest advantages, let us resolve to test the method, and if it contains wheat, garner it; if chaff, cast it to the winds. —*Proceedings of Colorado State Dental Association.*

